# Characterisation of novel functionality within the *Blastocystis* tryptophanase gene

**DOI:** 10.1371/journal.pntd.0009730

**Published:** 2021-09-07

**Authors:** Steven Santino Leonardi, Feng-Jun Li, Melissa Su-Juan Chee, John Anthony Yason, Hui Yi Tay, John Yu-Shen Chen, Eileen Yiling Koh, Cynthia Ying-Xin He, Kevin Shyong-Wei Tan

**Affiliations:** 1 Laboratory of Molecular and Cellular Parasitology, Healthy Longevity Translational Research Programme and Department of Microbiology and Immunology, Yong Loo Lin School of Medicine, National University of Singapore, Singapore; 2 Department of Biological Sciences, Faculty of Science, National University of Singapore, Singapore; 3 Infectious Diseases Translational Research Programme and Department of Microbiology and Immunology, Yong Loo Lin School of Medicine, National University of Singapore, Singapore; 4 Institute of Arts and Sciences, Far Eastern University, Manila, Philippines; University of Tokyo, JAPAN

## Abstract

In recent years, the human gut microbiome has been recognised to play a pivotal role in the health of the host. Intestinal homeostasis relies on this intricate and complex relationship between the gut microbiota and the human host. While much effort and attention has been placed on the characterization of the organisms that inhabit the gut microbiome, the complex molecular cross-talk between the microbiota could also exert an effect on gastrointestinal conditions. *Blastocystis* is a single-cell eukaryotic parasite of emerging interest, as its beneficial or pathogenic role in the microbiota has been a subject of contention even to-date. In this study, we assessed the function of the *Blastocystis* tryptophanase gene (*Bh*TnaA), which was acquired by horizontal gene transfer and likely to be of bacterial origin within *Blastocystis*. Bioinformatic analysis and phylogenetic reconstruction revealed distinct divergence of *Bh*TnaA versus known bacterial homologs. Despite sharing high homology with the *E*. *coli* tryptophanase gene, we show that *Blastocystis* does not readily convert tryptophan into indole. Instead, *Bh*TnaA preferentially catalyzes the conversion of indole to tryptophan. We also show a direct link between *E*. *coli* and *Blastocystis* tryptophan metabolism: In the presence of *E*. *coli*, *Blastocystis* ST7 is less able to metabolise indole to tryptophan. This study examines the potential for functional variation in horizontally-acquired genes relative to their canonical counterparts, and identifies *Blastocystis* as a possible producer of tryptophan within the gut.

## Author summary

*Blastocystis* is a genus of microscopic parasites able to infect the human colon. Whether it plays a role in disease has been a topic of debate in the scientific community since its discovery. Some evidence links infection with the parasite to an increase in biodiversity in gut bacteria, which is associated improvements in health. Other evidence links *Blastocystis* to symptoms of the common gut disease irritable bowel syndrome, or IBS. This study shows that *Blastocystis* interacts with the amino acid tryptophan in an unexpected manner, which may shed light how it might cause disease. We show that it uses an enzyme known as tryptophanase in reverse–in other organisms, tryptophanase converts tryptophan into another molecule known as indole, while *Blastocystis* converts indole to tryptophan. Cells in the gut called enterochromaffin cells convert tryptophan into the neurotransmitter serotonin. This molecule can influence the behaviour of the gut, and has been linked to symptoms of IBS. We believe that, under some circumstances, an infection with *Blastocystis* may cause an increase in tryptophan in the gut, which is then converted to serotonin. The excess serotonin would then cause symptoms of the disease.

## Introduction

*Blastocystis* is a highly prevalent stramenopilic parasite of the gastrointestinal system, estimated to be present in over one billion humans worldwide [1], along with a wide variety of mammalian, reptilian, amphibian, and avian hosts [2]. *Blastocystis* strains are classified into subtypes (ST) of the *Blastocystis* genus [3], rather than species. Genomic and ribosomal sequencing have identified 17 *Blastocystis* subtypes, designated ST1 through ST17 [4]. Of these, ST1 through 9 are found in humans [5]. Since 2013, eight new subtypes have been described, but their validity is disputed [6]. Complete genomic sequencing data is available for ST1, 4, and 7. ST7, used in this paper, is classified into various isolates, denoting their clinical origin [7]. This study focuses on subtype 7, isolate B, referred to as ST7-B.

Until recently, *Blastocystis* was assumed to be a commensal, as evidence of direct association with disease was elusive. This view is gradually shifting, as mounting clinical evidence links *Blastocystis* infection with higher rates of generic gastrointestinal symptoms, including diarrhoea, constipation, nausea, and abdominal pain [8,9], often characterised under the umbrella term ‘irritable bowel syndrome’ (IBS). IBS is a collective term for idiopathic symptoms of gastrointestinal dysfunction, including bloating, altered stool morphology, and abdominal pain. IBS is estimated to afflict between 7–16% of the population of the United States each year, and may be responsible for up to USD $1 billion in medical fees overall, as well as 10–15 Disability-Adjusted Life Years per patient [10]. Originally seen as a single disease, evidence now suggests that IBS is a group of disparate disorders with highly similar presentation.

Recent research has demonstrated the pathogenic potential of certain *Blastocystis* subtypes. Ajjampur *et al*. [11] linked *Blastocystis* with mucosal sloughing and epithelial damage in the large intestine of infected pigs, and previous work in our lab identified subtype ST7-B as being capable of inhibiting the growth of beneficial human gut microbiota *in vitro* and *in vivo*, as well as causing tissue damage to an *ex vivo* mouse explant model [7,12].

Through phylogenetic reconstruction, approximately 2.5% of the *Blastocystis* ST1 genome has been identified as originating via horizontal gene transfer (HGT) [13]. While this may seem as low compared to prokaryotic microorganisms (9.6% in *E*. *coli*; 14.5% in *M*. *tuberculosis* [14]), it is within the expected range for eukaryotes. Danchin *et*. *al* [15] note the extreme variability present in existing estimates for eukaryotic organisms. 2.5% of the *Blastocystis* genome amounts to approximately 167 genes originating from a variety of organisms, including some prokaryotic and mammalian. One example of a horizontally-acquired *Blastocystis* gene is tryptophanase (TnaA). TnaA is a prokaryotic enzyme responsible for converting tryptophan into indole, a compound capable of inducing changes in gene expression and behaviour between bacteria [16]. The tryptophan digestion pathway produces an array of secreted signalling molecules that can influence the host and other gut microbiota [17]. In light of the existing evidence suggesting *Blastocystis* may play a role in intestinal disorders, we investigated the role of this gene in order to understand its possible effect on the gut microbiota.

In this study, we investigated the properties and function of *Blastocystis* TnaA (*Bh*TnaA) from the previously reported pathogenic *Blastocystis* variant ST7-B [12]. We show that canonical *E*. *coli* K12 TnaA has limited homology with *Bh*TnaA, while the gene is highly conserved between *Blastocystis* ST 1, 4, and 7. We also showed that purified ST7-B *Bh*TnaA preferred the reverse reaction of TnaA, in which indole is converted to tryptophan. Based on our results, a ‘tryptophan cycle’ is proposed to exist within the gut micro-environment, in which *E*. *coli* and other tryptophan digesters of the gut microbiome produce indole that is then utilised by or converted to tryptophan by *Blastocystis*.

## Materials and methods

### Ethics approval and consent to participate

Human *Blastocystis* isolate ST7-B was acquired from a patient at the Singapore General Hospital in the early 1990s, before the Institutional Review Board was established at the National University of Singapore (NUS). Samples taken at the time were anonymized and do not contain any patient identifiers.

### Cell strain and culture

An axenized *Blastocystis* isolate of ST7-B cells was used in this study. Cells were cultured in pre-reduced Iscove’s Modified Dulbecco’s medium (IMDM) (Thermo Fisher Scientific, USA) supplemented with 10% horse serum (Gibco, USA) at 37°C. The culture tubes were maintained inside sealed anaerobic jars with an anaerobic gas pack (Oxoid, UK). Cultures were subcultured every 3–4 days.

### Bioinformatic analysis

The protein sequence of bacterial TnaA was aligned against the *Blastocystis* protein database using the standard NCBI protein blast (*blastp*) algorithm (https://blast.ncbi.nlm.nih.gov/Blast.cgi?PAGE=Proteins). The multi-alignment of TnaA orthologues in *Blastocystis* ST1 NandII, ST4, and ST7 strains against *E*. *coli* K12 (Table 1) was performed using Jalview (version 2.11.0) [18].

**Table 1:** Tryptophanase Sequences for Bioinformatic Analysis.

Tryptophanase Sequence	Reference
*E*. *coli* K12	Deeley & Yanofsky 1981 [20] Uniprot Accession No.: P0A853
*Blastocystis* ST1	Eme *et*. *al*. 2017 [13] Genbank Accession No.: LXWW00000000.1
*Blastocystis* ST4	Wawrzyniak *et*. *al*. 2015 [21] Genbank Accession No.: JPUL02000000
*Blastocystis* ST7	Denoeud *et*. *al*. 2011 [22] BioProject Accession No.: PRJNA263384

Phylogenetic analysis of TnaA from protozoan parasites (*Blastocystis*, *Entamoeba* and *Trichomonas*), Gammaproteobacteria, Alphaproteobacteria and Clostridia were conducted using the PHYLIP-based PhyML3.0 server (http://www.atgc-montpellier.fr/phyml/), with alignment via the Neighbor-Joining method [19].

The predicted 3-D protein structure of *Blastocystis* ST7-B TnaA was constructed using the Protein Homology/analogy Recognition Engine V2.0 (Phyre2) server (http://www.sbg.bio.ic.ac.uk/phyre2/html/page.cgi?id=index) using the default parameters.

### Tryptophan assay (Figs 4 and 5)

The total tryptophan in each reaction was determined using the Tryptophan Assay Kit (Sigma-Aldrich, Inc., USA) as per manufacturer’s protocol. In brief, after layering the reaction mixture [200 mM potassium buffer (pH 7.5), 0.165 mM pyridoxal-50-phosphate (PLP), 0.2 mM reduced glutathione, 0.25 mg/ml bovine serum albumin, 10 mg/ml purified TnaA, and varying concentrations of indole] with 100ml of toluene, it was pre-warmed for 5 min at 37°C. After a 10 min incubation period, 100μl of the supernatant from each reaction was transferred to a new PCR tube, with 20μl of tryptophan condenser added to each sample, mixed and incubated at room temperature for 2 min. A volume of 20μl TRP catalyst was added to each sample, mixed, and the sample tubes incubated at 105°C for 60 min. The contents in the tube were allowed to settle to the bottom of the tube prior to transferring 130μl of sample supernatant from each tube into a 96-well plate. Fluorescence intensity was measured at λ_ex_ = 370 nm and λ_em_ = 440 nm using the Hidex Sense multimode microplate reader (Hidex Oy, Finland). The amount of tryptophan in each reaction mixture was calculated from a standard curve. Kinetic parameters were computed from a Hanes-Woolf transformation of the Michaelis–Menten equation. Data were obtained from three independent experiments.

### Tryptophan assay (Figs 7 and 8)

The total tryptophan in culture media was determined using the Tryptophan Assay Kit (BioVision, USA) as per manufacturer’s protocol. A volume of the media from each tube was transferred to a 1.5mL Eppendorf tube and made up to 110μl using ddH_2_O. Tryptophan condenser (20 μl) was added to each sample, mixed and incubated at room temperature for 2 min. A volume of 20μl tryptophan catalyst was added to each sample, mixed, and the sample tubes incubated at 105°C for 60 min. A sample control tube was also prepared following the same protocol, without the addition of the catalyst. The contents in the tubes were allowed to settle to the bottom prior to transferring 130μl of sample supernatant from each tube into a 96-well plate. Fluorescence intensity was measured at λ_ex_ = 370 nm and λ_em_ = 440 nm using the Tecan Infinite M200 Pro multimode microplate reader (Tecan Life Sciences, Switzerland). Sixteen separate measurements were taken from different locations within each well. The amount of tryptophan in each well was calculated from a standard curve as per manufacturer protocol. Three independent replicates were performed.

### Indole assay

The total indole in culture media was determined using the Indole Assay Kit (Assay Genie, Ireland) as per manufacturer’s protocol. A volume of 100μl of the media from each tube was transferred to a 96-well plate before being combined with 100μl of the Working Reagent. Absorbance was then measured at 400nm using the Tecan Infinite M200 Pro multimode microplate reader (Tecan Life Sciences, Switzerland). Sixteen separate measurements were taken from different locations within each well. The amount of indole in each well was then calculated from a standard curve as per manufacturer protocol. Three independent replicates were performed.

### Expression of GST-tagged *Blastocystis* tryptophanase

The coding region of *Blastocystis* tryptophanase was amplified from a *Blastocystis* ST7-B cDNA library using the following primers:

TnaA-F: cgggatccATGCCTTTCGTTCCTGCATCT

(*BamH*I restriction site underlined)

TnaA-R: ccgctcgagTTACTTCTCCTCGGGAATGGG

(*Xho*I restriction site underlined)

The PCR product was digested by *BamH*I and *Xho*I and ligated into *Bam*HI/*Xho*I digested pGEX-6p-1 vector. The construct was then transformed into an *E*. *coli* BL21 strain for expression. To increase the solubility of expressed TnaA, the transformed *E*. *coli* BL21 culture (measured at OD600 of approximately 0.4) was heat-shocked at 42°C for 10 min, followed by incubation for a further 20 min at 37°C. Subsequently, the transformed *E*. *coli* BL21 cells were incubated on ice for 30 min, cultivated at 37°C for 20 min, and induced with 0.1 mM IPTG for 20 hrs at 20°C.

The TnaA deactivation double mutant (K322A/K323A, R529A/H530A/Q532A) was constructed by first creating the KA/KA mutation through overlap-extension PCR with two primer pairs: TnaA-F and TnaA-R2 (CACCAGACCGTCAGCTGCCAGGGACATGGTGAAG); TnaA-F2 (CTTCACCATGTCCCTGGCAGCTGACGGTCTGGTG) and TnaA-R. A complementary sequence bridging the two lysines was introduced at the 3’- and 5’-end of each fragment, and the sequence of each lysine was changed to an alanine sequence. After overlap-extension, the fused fragment was amplified with primers TnaA-F and TnaA-R and digested by *BamH*I and *Xho*I. The digested fragment was then cloned into pGEX-6P-1 vector and the resultant vector amplified with primer pair:

TnaA-1 (GCCGAGCTGCGCCTGGCAGCATTCGCATCCGGAATTGCGCCT) and TnaA-2 (AGGCGCAATTCCGGATGCGAATGCTGCCAGGCGCAGCTCGGC), followed by *Dpn*I digestion. The digested vector was transformed into *E*. *coli* BL21 to obtain the final double mutant vector and the second mutation was verified by DNA Sanger sequencing with the following primer pairs: TnaA-RT-F: ACGAGGAGCTGATCAAGGAG; TnaA-RT-r: TACTTCACCTTGTCGCCAGT.

### Purification of GST-tagged tryptophanase

The wildtype and double-mutants of TnaA expression vectors were transformed into *E*. *coli* BL21 strain. The TnaA recombinant proteins were purified by washing and resuspending the transformed *E*. *coli* BL21 with PBS, followed by sonication for cell lysate preparation. The GST-tagged *Blastocystis* TnaA was bound to Glutathione Sepharose 4B beads (GE Healthcare, USA) following the purification protocol provided by the manufacturer.

### On-beads digest with PreScission protease and dialysis

The GST-free TnaA was released from the Glutathione Sepharose 4B beads by digestion with PreScission protease (GE Healthcare, USA). Briefly, the fusion protein-bound matrix was washed with 10 bed volumes of Cleavage Buffer at 5°C and residual buffer removed. For each ml of washed Glutathione Sepharose bed volume, 80 units of PreScission Protease was mixed with 960 μl of Cleavage Buffer at 5°C. The mixture was subsequently added to the fusion protein-bound Glutathione Sepharose, gently resuspended and incubated at 5°C for 4 hours. Eluate was collected by centrifugation of the bulk Glutathione Sepharose matrix at 500*x* g for 5 minutes and subjected to a second round of chromatography to remove the residual protease.

### TnaA enzymatic assay

L-Tryptophan degradation by purified TnaA was examined by measuring indole formation [23,24]. Briefly, after layering the reaction mixture [200 mM potassium buffer (pH 7.5), 0.165 mM pyridoxal-50-phosphate (PLP), 0.2 mM reduced glutathione, 0.25 mg/ml bovine serum albumin, 10 mg/ml purified TnaA, and several concentrations of L-tryptophan] with 100 ml of toluene, the reaction mixture was pre-warmed for 5 min at 37°C. After a 10 min incubation period, the reaction was terminated by the addition of 1 ml of Ehrlich’s reagent. The supernatant was examined spectrophotometrically at 568nm using a Hidex Sense multimode microplate reader (Hidex Oy, Finland). The amount of indole in each reaction mixture was calculated from a standard curve. Kinetic parameters were computed from a Hanes-Woolf transformation of the Michaelis–Menten equation. Data were obtained from three independent experiments.

### Flow cytometry and cell counting

3*10^6^ *Blastocystis* ST7-B cells were incubated at 37°C for 24 hours in PBS supplemented with varying concentrations of indole or tryptophan. The contents of the tubes were then vortexed once and washed in PBS three times (centrifugation 4500g, 5 minutes between washes). One sample was heat-killed at 80°C for 15 minutes before all samples were stained with 20μg mL^-1^ propidium iodide (Thermo-Fisher, USA) for 15 minutes. Standard flow cytometry was then performed using a BD LSRFortessa flow cytometer (BD Biosciences, USA). Results were processed using FCSAlyzer v0.9.22-alpha (https://sourceforge.net/projects/fcsalyzer/). For gating strategy, see S1 Fig. Prior to flow cytometry, 2μL of the cells were collected and diluted 100X. The dilutions were later counted on a Glasstic microscope slide (KOVA, USA).

### *E. coli* strains

BL21 *E*. *coli* was used for isolated *Bh*TnaA expression (Figs 4 and 5). MG1655 *E*. *coli* cells were used for co-culture experiments with *Blastocystis* (Figs 7 and 8). Details and descriptions of the strain are listed in Table 2.

**Table 2:** *E*.*coli* strains used and generated in this study.

Strain name	Genotype description	Reference
MG1655 / JCEC- 2640	F- lambda- *ilvG*- *rfb*-50 *rph*-1	Blattner *et al*. 1997 [25]
BL21	F- ompT gal dcm lon hsdSB(rB–mB–) [malB+]K-12(λS)	Jeong *et al*. 2006 [26]

### Co-culture of *Blastocystis* and *E*. *coli*

For the co-culture experiments, 10^7^ *Blastocystis* ST7-B were incubated with 10^7^ *E*.*coli* MG1655. The cells were incubated in either PBS, indole-supplemented PBS or PBS supplemented with tryptophan. After 24 hours, tryptophan levels were determined via the Tryptophan Assay Kit (BioVision, USA) and indole levels measured using the Indole Assay Kit (Assay Genie, Ireland).

### Statistical analysis

All statistical analysis was done using unpaired, two-tailed Student’s t-tests in GraphPad Prism software version 6, with the exception of Figs 4D, 5B, 7 and 8, which used two-way ANOVA tests done in the same program.

## Results

### Tryptophanase is conserved between *Blastocystis* and *E*. *coli* to a limited degree, with *Bh*TnaA containing a loop not present in the TnaA-like superfamily

**Fig 1:**
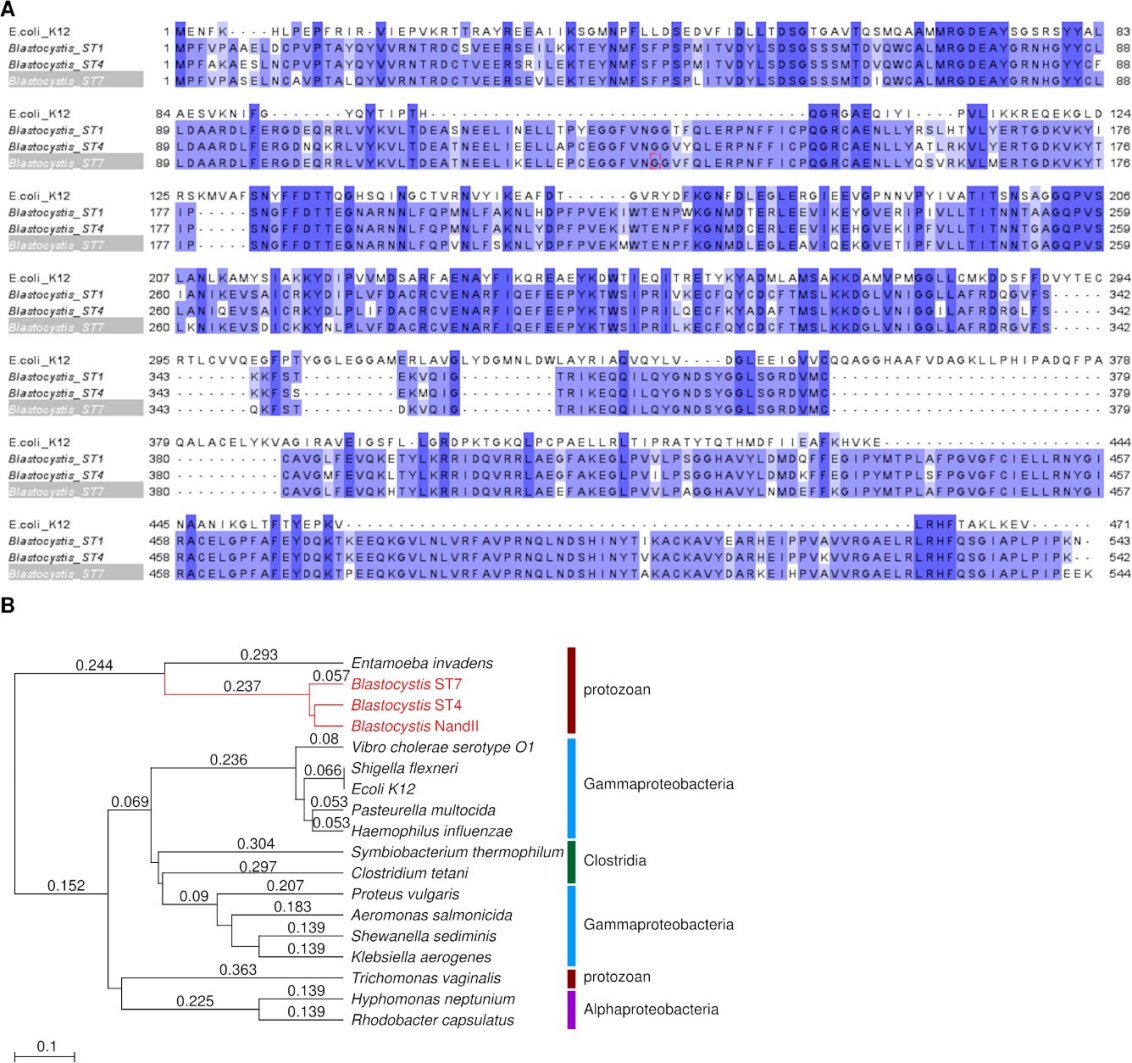
*Blastocystis* tryptophanase is conserved across subtypes. A) BLAST multiple sequence alignment of the protein sequence of the most common isoform of TnaA from *E*. *coli* K12 and *Blastocystis* subtypes ST4, ST7, and ST1. Regions of homology between sequences are highlighted blue. Generated using Jalview [18] B) Phylogenetic tree of TnaA-expressing microorganisms, generated using PhyML [19]. Branches exclusive to *Blastocystis* subtypes are highlighted in red. Numerical values represent substitutions per site. For sequences used, see Table 1.

**Fig 2:**
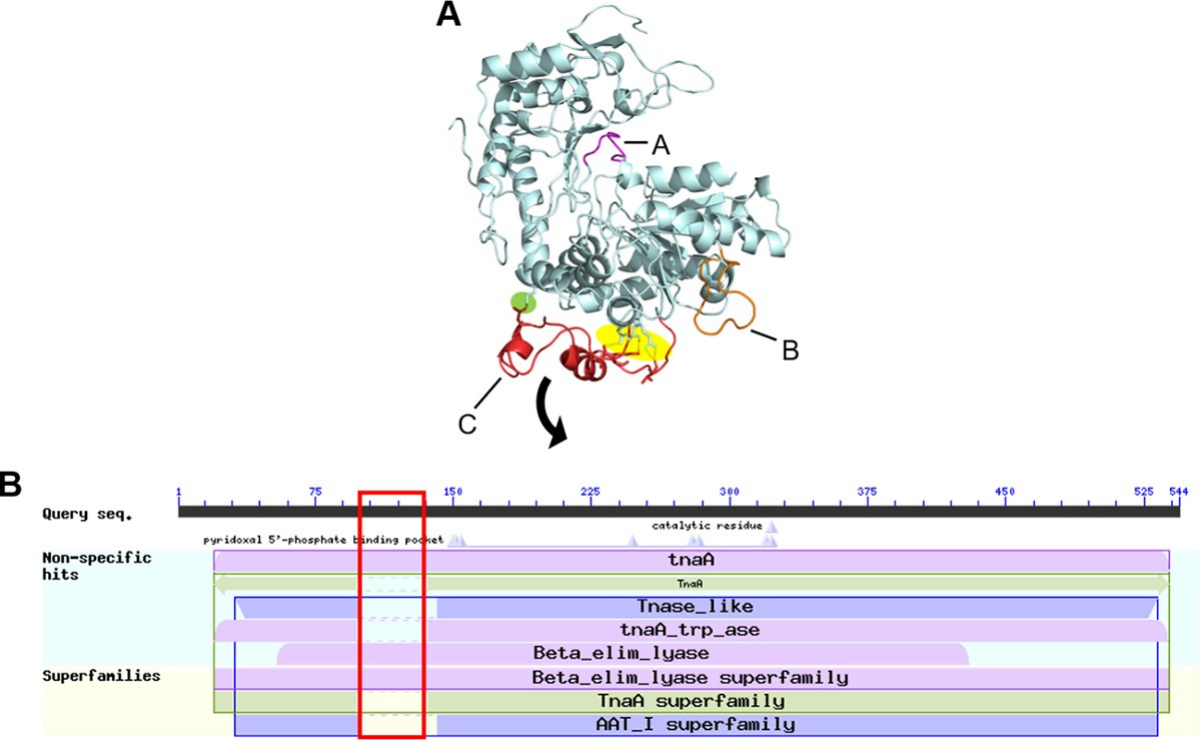
*Bh*TnaA contains unique regions not present in other tryptophanase proteins. A) Tertiary structure of ST7-B *Bh*TnaA generated via PHYRE2. Light blue represents area structurally similar to *E*. *coli* K12 TnaA. Domains A (purple), B (orange), and C (red) are loops not present in the tertiary structure of *E*. *coli* K12 TnaA. Highlighted in yellow is a putative hinge structure, while highlighted in green is a polar bond whose disruption is necessary for the functioning of the hinge. B) Output from the NCBI Conserved Domain Database (CDD)[27] when the protein sequence of ST7-B *Bh*TnaA is provided. Pink represents sequences from the beta-eliminating lyase superfamily, green represents the TnaA superfamily, and blue represents TnaA-like sequences, including the AAT I superfamily. The red box outlines the approximate location of Domain C within the results.

TnaA expression is found in a wide range of eukaryotes and prokaryotes, but its cross-species homology between *Blastocystis* and other microorganisms is unknown. To assess this, a multiple sequence alignment of the TnaA protein sequence from two TnaA-expressing gammaproteobacteria (*E*. *coli*) and the three sequenced *Blastocystis* subtypes (ST4, ST7-B, and ST1) was constructed (Fig 1A). For sequences used, see Table 1. Only 35% of the TnaA protein was conserved between *Blastocystis* and *E*. *coli*, with higher homology between the intra-gammaproteobacterial and *Blastocystis* spp. A phylogenetic tree based on the TnaA sequences was then constructed to evaluate the evolutionary distance between other known prokaryotic TnaA and the *Blastocystis* subtypes (Fig 1B). The tree showed considerable divergence between the Alpha/Gamma-Proteobacteria / Clostridia group and the protistan group to which the *Blastocystis* subtypes clustered. This supported the low orthology of the aligned TnaA sequences previously observed. Based on available information, a putative tertiary protein structure of the *Bh*TnaA was constructed (Fig 2A), rendering high similarities to the bacterial homolog despite low consensus at the protein level. Three domains–indicated as A, B and C–were further identified within *Bh*TnaA that were not present in the *E*. *coli* TnaA. Domain C is attached to the main body of the protein by two amino acid strands alone, indicating a possible protein hinge. Four polar bonds connect Domain C to the main body, three of which are at the hinge. A single polar bond holds the domain ‘closed’, as shown in the diagram. When aligned against the NCBI Conserved Domains Database (CDD)[27], the approximate area of domain C was shown to be absent from all members of tryptophanase and tryptophanase-like superfamilies and present in the beta-eliminating lyase superfamily. Unexpectedly, the consensus TnaA sequence from NCBI did contain a homolog of Domain C, while consensus sequences from other sources did not.

### *Blastocystis* TnaA can metabolise tryptophan

**Fig 3:**
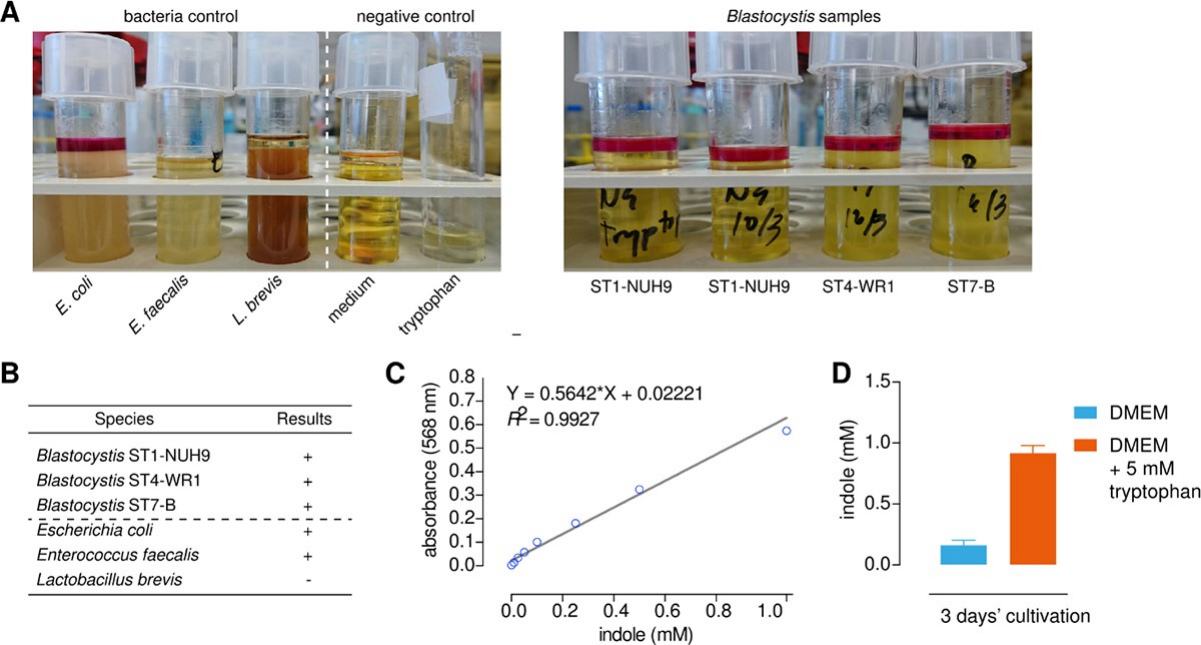
*Blastocystis* is capable of producing indole from tryptophan. A) Indole test comparing indole production capability of *Blastocystis* subtypes (right-hand image) with bacterial and negative controls (left-hand image). Positive result for indole is indicated by the formation of a rosindole layer when combined with Kovac’s Reagent, for example in the *E*. *coli* control tube. B) Tabulation of the bacterial control and *Blastocystis* subtype results from Fig 2A, where + = indole-positive and— = indole-negative. C) Standard curve of the absorbance at 568nm of the rosindole layer created by reacting different quantities of indole with Kovac’s Reagent. D) Effect of increasing tryptophan concentration on indole production by *Blastocystis* ST7-B after a three-day cultivation period. Indole concentration was determined by measuring the absorbance of the rosindole layer, and applying the equation generated in Fig 2C.

Given the high degree of dissimilarity between the *E*. *coli* and *Blastocystis* TnaA sequences, the indole test was used to assess whether *Bh*TnaA was capable of converting tryptophan to indole. A positive result of this test was characterised by the production of a pink layer of rosindole when Kovac’s reagent [28] was introduced into culture. *Blastocystis* ST1, 4, and 7 generated a layer comparable to that of the positive control *E*. *coli*, demonstrating that *Bh*TnaA possesses TnaA-enzymatic capabilities (Fig 3A and 3B). A standard curve for indole concentration was prepared by measuring the absorbance of the rosindole layer when supplementing media with varying concentrations of tryptophan, showing a linear relationship with high R^2^ value of 0.9927 (Fig 3C). When DMEM media was supplemented with an additional 5mM tryptophan, *Blastocystis* ST-7B cultures showed an approximate three-fold increase in indole concentration after three days culture (Fig 3D).

### Purified *Blastocystis* tryptophanase behaves similarly to that of *E*. *coli*, but with low reaction *K*_*m*_

**Fig 4:**
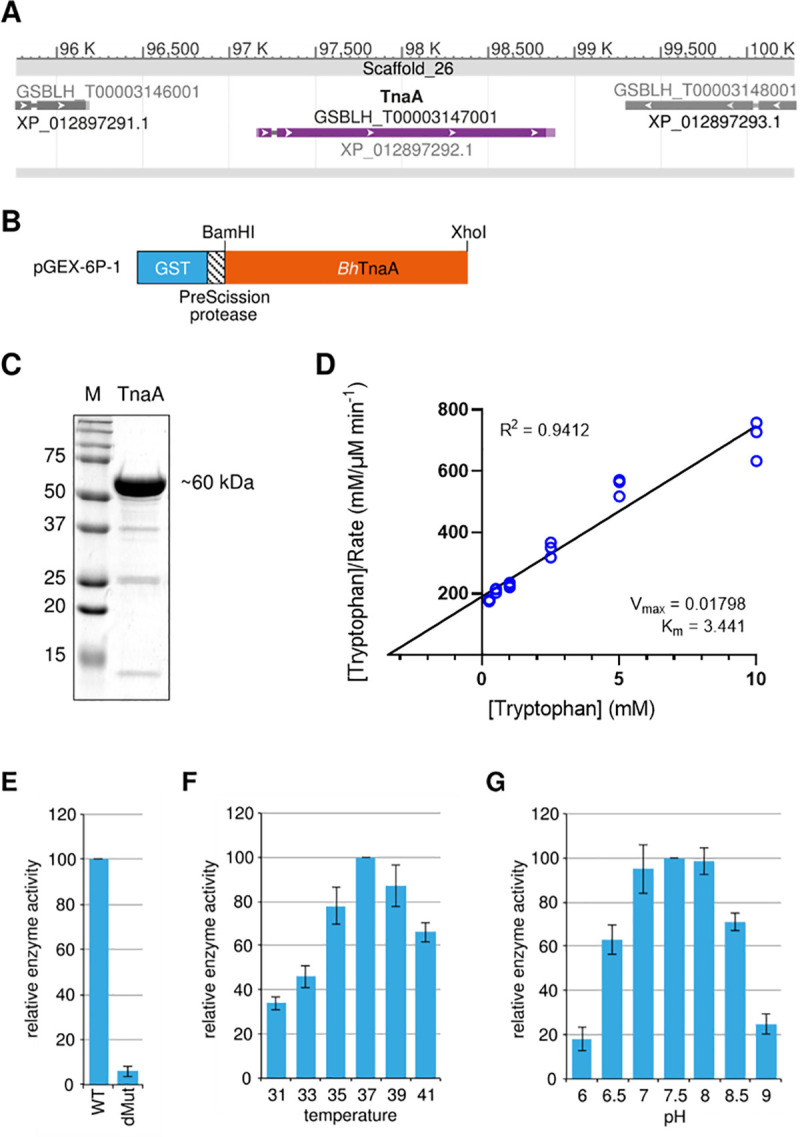
*Bh*TnaA functions ideally at human body temperature and pH. A and B) Simplified map of the *Bh*TnaA insert within the pGEX-6P-1 vector. *Bh*TnaA is tagged with GST, with a PreScission protease sequence for cleavage of the tag. C) Western blot of the purified and cleaved *Bh*TnaA isolate. Used to demonstrate validity of the protein purification process. The TnaA band is equivalent to the mathematically-derived expected size of the protein. D) Hanes-Woolf plot generated via supplementing purified *Bh*TnaA with increasing concentrations of tryptophan, and determining quantity of produced indole via absorbance (as in Fig 2C). V_max_ is in units of μM product generated min^-1^. K_m_ is in units of mM substrate. Curve calculated for the mean of the three data points shown at each tryptophan concentration. E) Effect of *Bh*TnaA inactivation (dMut) on indole production. Production calculated as rosindole layer absorbance post-tryptophan supplementation and 72 hrs incubation. F and G) Optimal temperature and pH for purified *Bh*TnaA activity. Experiment identical to Fig 3E with variation of either temperature during incubation, or pH of media. Error bars were calculated using Student’s t-test.

Further bioinformatic analysis revealed that BhTnaA loci was located on Scaffold 26 of the *Blastocystis* genome. The *BhTnaA* protein was isolated and purified for subsequent experiments through the generation of a PreScission-tagged *Bh*TnaA insert that was embedded into a pGEX-6P-1 vector. PreScission is a Glutathione-S-Transferase (GST) tag combined with an HRV-3C protease sequence (Fig 4B). Using this plasmid, the tagged protein was purified from transformed *E*. *coli* colonies, the insert was extracted via restriction digest, and the *Bh*TnaA sequence cleaved from the PreScission tag. The purified protein was verified by size via Western Blot (Fig 4C). Using the purified protein, some basic properties of *Bh*TnaA were established. We generated a Hanes-Woolf plot to investigate the kinetics of the *Bh*TnaA forward reaction (where tryptophan is converted to indole) (Fig 4D). This tryptophan-to-indole plot showed the reaction to be inefficient, requiring the concentration of tryptophan substrate to exceed 3mM before K_m_ was reached. A K322A/K323A, R529A/H530A/Q532A double mutant (Fig 4E) was generated to demonstrate the abolishment of enzyme activities and support the absence of endogenous contamination. When enzymatic activity was abolished, indole production ceased, indicating that *Bh*TnaA was the sole producer of indole within the *Blastocystis* proteome. Enzymatic activity was greatest at 37°C and between pH 7 and 8 (Fig 4F and 4G). These optimal conditions are as expected for the typical living environment of *Blastocystis* within the colon.

### *Bh*TnaA exhibits a preference for its reverse reaction

**Fig 5:**
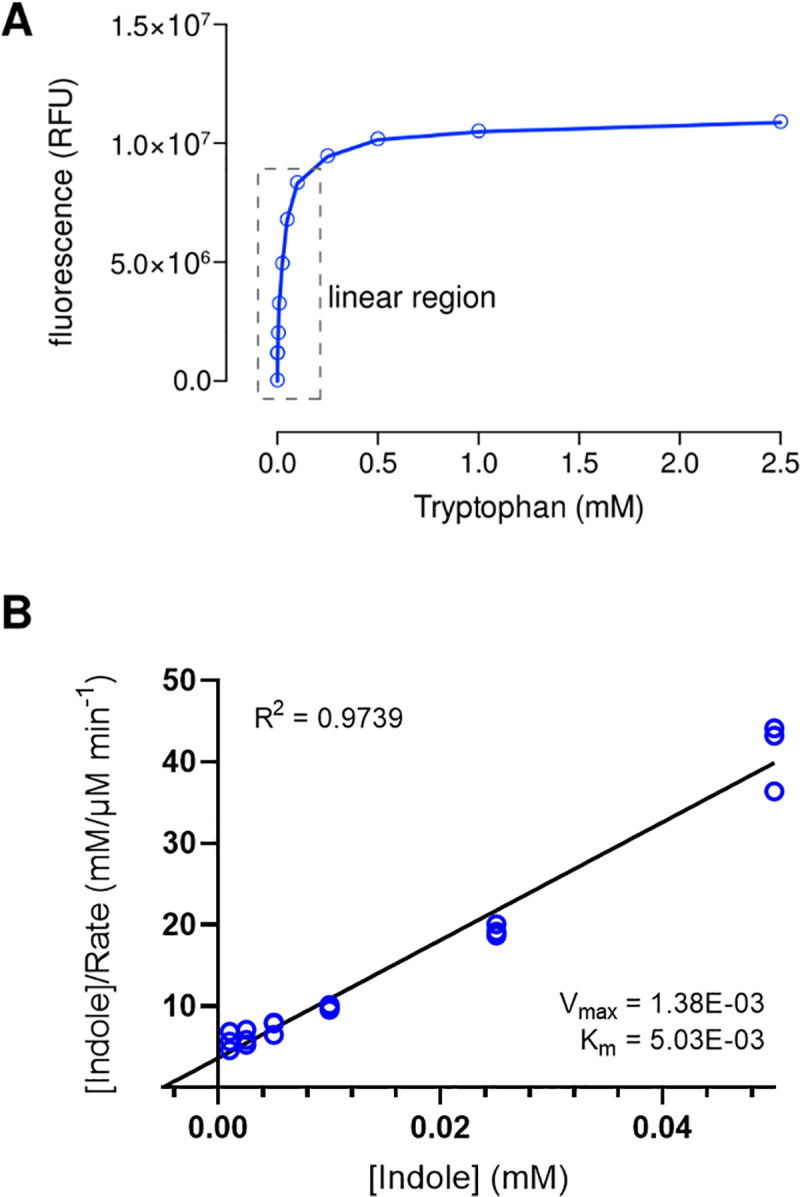
*Bh*TnaA preferentially performs the tryptophanase reverse reaction. A) Standard curve of fluorescent signal generated by differing concentrations of tryptophan in solution. Tryptophan was dissolved in DMEM media, which was then assessed for fluorescence at 370 and 440nm. B) Hanes-Woolf plot generated via supplementing purified *Bh*TnaA with increasing concentrations of indole, and determining quantity of produced tryptophan via fluorescence. Fluorescence was measured as in Fig 4A. V_max_ is in units of μM product generated min^-1^. K_m_ is in units of mM substrate.

Given the low affinity of *Bh*TnaA towards its forward reaction (where tryptophan is converted to indole), investigations were carried out to determine whether the reverse reaction (i.e. the conversion of indole to tryptophan) occurred at a greater affinity or at a preferred rate. Experiments similar to those in our earlier investigation of tryptophan conversion to indole were carried out, but indole was provided as substrate and tryptophan output was determined using fluorometric analysis. The K_m_ of this reaction was 5.03*10^−3^ (Fig 5B), indicating a far higher affinity of the enzyme for the substrate when compared to the forward reaction.

### Tryptophan promotes *Blastocystis* survival and growth

**Fig 6:**
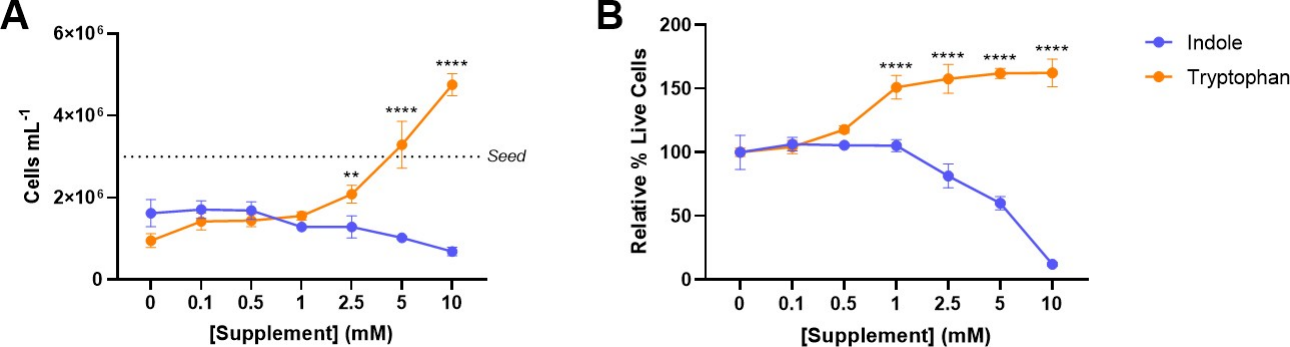
Indole and tryptophan have opposing effects on *Blastocystis* viability. 3*10^6^ *Blastocystis* ST7-B were seeded in PBS supplemented with increasing concentrations of indole or tryptophan. A) Remaining number of live cells as determined by manual counting using a hemocytometer. *Seed* line indicates initial number of cells. B) Relative remaining proportion of live cells as determined by propidium iodide stain. Significance was calculated using a two-way ANOVA, with ** = p<0.01 and **** = p<0.0001. N = 3 for all data points.

To determine the effects of indole and tryptophan on *Blastocystis* cell growth, concentrations of substrate between 0 – 10mM were added exogenously to the culture media. Increasing concentration of tryptophan in culture media increased the number of cells present and the proportion of live cells following the 24hr culture period. Indole demonstrated the opposite effect, reducing both the number and viability of cells (Fig 6A and 6B). This result was also observed when the cells were cultured in IMDM-HS, rather than PBS (S2 Fig).

### *E*. *coli* affects *Blastocystis’* ability to synthesise tryptophan

**Fig 7:**
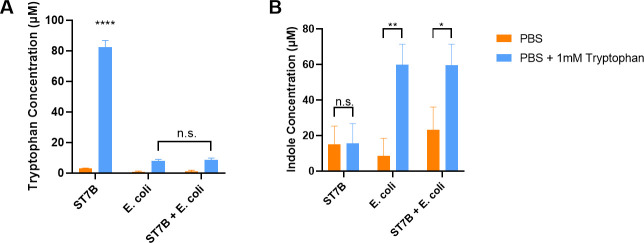
*Blastocystis* ST7-B does not convert tryptophan to indole readily. A) Graph of tryptophan concentration post-24-hour incubation period when culturing *Blastocystis* and *E*. *coli* in PBS supplemented with tryptophan. B) Graph of indole concentration in the same cultures. Significance levels were calculated using a two-way ANOVA, with * = p<0.05, ** = p<0.01, and **** = p<0.0001. N = 3 for all columns.

**Fig 8:**
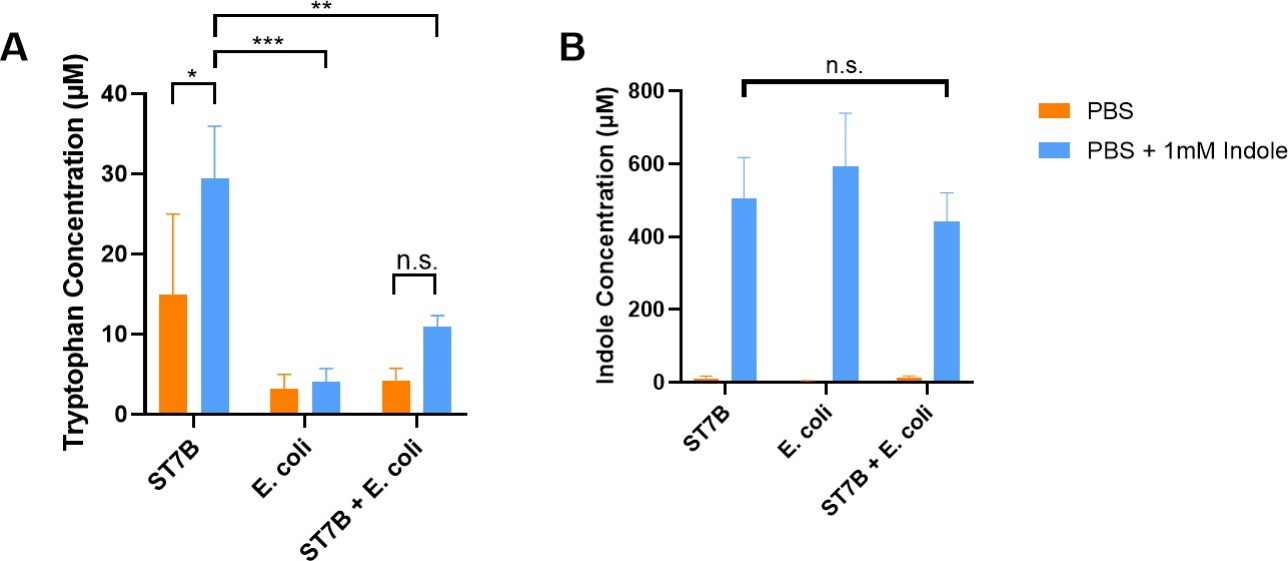
*Blastocystis* ST7-B produces tryptophan when supplemented with indole. A) Graph of tryptophan concentration post-24-hour incubation period when culturing *Blastocystis* ST7 and *E*. *coli* in PBS supplemented with indole. B) Graph of indole concentration in the same cultures. Significance levels were calculated using a two-way ANOVA, with * = p<0.05, ** = p<0.01, and *** = p<0.001. N = 3 for all columns.

We investigated our hypothetical ‘tryptophan cycle’ further by co-culturing *E*. *coli* and *Blastocystis* ST7 in PBS minimal media supplemented with either tryptophan or indole. Following the 24hr incubation period, we measured the remaining concentration of both indole and tryptophan. As shown in Fig 7A, *Blastocystis* alone in culture was unable to metabolise the supplemented tryptophan, leaving the post-incubation concentration up to ~10x greater than supplemented *E*. *coli*. Supplementation of *E*. *coli* with tryptophan, resulted in a lower post-incubation tryptophan concentration, as is expected for a known tryptophan metaboliser. When *Blastocystis* and *E*. *coli* were co-cultured, the post-incubation tryptophan concentration in the medium was reduced in a similar manner to *E*. *coli* alone. When *Blastocystis* was cultured in indole-supplemented medium, a significantly higher concentration of tryptophan was measured (Fig 8A) demonstrating the indole-metabolising activity of the *Bh*TnaA gene. Conversely, *E*. *coli* was unable to digest the supplemented indole efficiently as evident in the lack of increased tryptophan above the non-supplemented baseline level. When the two microorganisms were co-cultured, a small increase in tryptophan concentration was observed, however it was not statistically significant. In Fig 7B, *Blastocystis* ST7 does not synthesise indole in response to supplemented tryptophan, while *E*. *coli* does so. When the two were co-cultured, production of indole was still observed, most likely due to *E*. *coli* activity. Conversely, no significant differences were observed between indole levels in indole-supplemented medium after the incubation period, regardless of whether *Blastocystis*, *E*. *coli*, or both were present (Fig 8B).

### *Blastocystis* and prokaryotic microbiota may metabolise tryptophan in a cyclic manner

**Fig 9:**
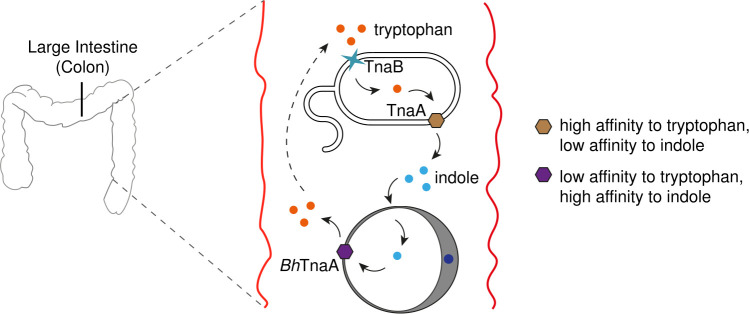
Tryptophan and Indole may exist cyclically in a *Blastocystis*-infected gut. Diagram of a hypothetical ‘tryptophan cycle’ between *E*. *coli* (upper) and *Blastocystis spp*. (lower) within the descending colon. Tryptophan is shown in orange, and indole is shown in blue. TnaA enzymes are depicted as hexagons, and *E*. *coli* tryptophan permease (TnaB) is depicted as a blue star. Extracellular tryptophan is taken up by *E*. *coli* using TnaB [43], and was processed into indole by TnaA before being secreted back into the environment. *Blastocystis* takes up and converts the indole back into tryptophan using *Bh*TnaA, before secreting it. This tryptophan can then be re-metabolised by *E*. *coli*.

Our results using *Blastocystis* cocultured with *E*. *coli* (Figs 7 and 8) suggest that *Blastocystis* TnaA may have high affinity for indole produced by the microbial components in the gut environment, using it as potential energy source and converting it to tryptophan, which is returned to the environment, creating a ‘tryptophan cycle’ (Fig 9). Depicted here is a hypothetical tryptophan cycle between *Blastocystis* and *E*. *coli*, in which *E*. *coli* synthesises indole, which in turn is used by *Blastocystis* to generate tryptophan.

## Discussion

Tryptophan, though an important amino acid in the human system, cannot be synthesised within the body and is usually obtained through diet [29]. The subsequent digestion of tryptophan into indole is a well-understood biochemical process that leads to the production of a number of gene expression-modulating compounds, including serotonin [30]. It also contributes to the chemical composition of the gut microbiome [31]. While some studies exist examining the presence of *Blastocystis* in clinical patients [8,9] or the pathogenic activity of a *Blastocystis* subtype [12], no research has yet investigated a molecular basis for *Blastocystis-*associated symptoms.

Our study investigates the gene responsible for tryptophan metabolism in *Blastocystis*, *Bh*TnaA. It was initially thought that *Bh*TnaA was functionally similar to its bacterial counterparts, whereby tryptophan was used as substrate to produce indole. However, protein and phylogenetic alignments (Fig 1A and 1B, respectively) together with structural modelling of the enzyme (Fig 2A) has shown that *Bh*TnaA exists in a markedly different form and is evolutionarily divergent from the canonical *E*. *coli* K12 TnaA. In fact, there are very few observed conserved regions of the TnaA protein sequence between *Blastocystis* and bacteria. Such differences could explain the unique tryptophan synthesis behaviour displayed by the enzyme.

The initial acquisition of *E*. *coli* TnaA by *Blastocystis* via HGT [13] is intriguing. It raises the question of the protein’s role within the organism prior to the eventual reversal of its activity. Gogarten and Townsend [32] hypothesised that the majority of HGT-acquired genes are neither deleterious or beneficial to their new host initially, instead having little to no effect on host fitness. These acquired genes would then evolve over time to gain function within the organism [32,33]. *Bh*TnaA is evidence of the latter stage of this process–there are currently no other known organisms with reverse-operating TnaA variants, suggesting this functionality developed within *Blastocystis* at a point in time after the transfer of the gene. This hypothesis also implies the existence of large numbers of horizontally-acquired genes with divergent functions from their evolutionary ancestors, although the incidence of such genes has not yet been fully investigated.

A closer look into the protein structure of *Bh*TnaA revealed significant differences compared to the canonical structure of *E*. *coli* TnaA. These differences were localised to three previously unreported domains, labelled A, B, and C. We hypothesise that Domain C in the tertiary structure of *Bh*TnaA (Fig 2A) may be involved in its unique interaction with tryptophan. Domain C of *Bh*TnaA is a sizeable region that is loosely bound to the main protein body, and is relatively unique among TnaA-like proteins. This suggests it may be capable of hinging away from the main body of the protein and admitting a substrate such as tryptophan. The single polar bond between N116 and K389 could be disrupted by the approach of the substrate, allowing for the conformational change. As this is the first observation of structural differences between *Blastocystis* and *E*. *coli* TnaA, especially with the presence of three previously unreported domains in the former, there is significant potential for further research. Future work can focus on manipulating these domains to identify whether there is a link between them and *Bh*TnaA’s tryptophan-binding capability.

There is a large degree of conservation of the *Bh*TnaA sequence between subtypes 1, 4, and 7 (Fig 1B), suggesting that many, if not all, of the other subtypes could also express a similar gene. The ability to convert indole to tryptophan is therefore highly valuable to *Blastocystis* and may play a critical role in supporting their survival in the gut. Both indole and tryptophan are critical compounds within and outside the gut microenvironment–indole is an intercellular signalling molecule [34], while tryptophan is the precursor to a number of critical metabolites, including serotonin and kynurenine [30].

When *E*. *coli* absorbs tryptophan, it can be metabolised in a variety of ways. Aside from being converted to indole, the tryptophan can also be decarboxylated to tryptamine, which is then secreted. Furthermore, the enzymes indoleacetamide monooxygenase (iaaM) and indoleacetamide hydrolase (iaaH) can serve to convert tryptophan into the immunotoxic ligand indole-3-acetic acid [17]. Indoleacetic acid can be again decarboxylated, converting it to 3-methyl indole (skatole). The function of indoleacetic acid is not well characterised outside of plants, where it is known as a promoter of plant growth and development [35], while skatole has been shown to present a pneumotoxic effect towards pigs [36]. These two compounds are both secreted by *E*. *coli*, and have clear potential to affect the health and the microbiome of the host organism–especially skatole, in the case of mammals. These compounds and pathways are likely to be present in *Blastocystis* as well as *E*. *coli*. The implications of reversing the tryptophan-to-indole activity of TnaA within the larger context of the entire pathway may extend to many, if not all, of the compounds produced by the pathway, creating the potential for unknown, possibly pathogenic effects within the host. Given our previous research linking *Blastocystis* ST7 with pathogenicity [12], the products of a ‘*Blastocystis* variant’ of the tryptophan metabolism pathway may shed light on some of the gastrointestinal symptoms associated with the organism.

Initially, we demonstrated that *Blastocystis* could convert tryptophan to indole (Fig 2). This suggested that TnaA from *E*. *coli* and *Blastocystis* functioned similarly despite their low sequence homology. Through enzyme kinetics (Figs 4D and 5B), it was shown that *BhTnaA* exhibited a marked preference for the reverse reaction of canonical TnaA, in which indole is converted to tryptophan. We also showed that *Bh*TnaA was the sole protein involved in the metabolism of tryptophan within *Blastocystis* (Fig 4E), and that the optimal conditions for the functioning of the enzyme were identical to that of the human colon, the habitat of the parasite (Fig 4F and 4G).

Fig 6A and 6B showed that, at concentrations of 2.5mM or greater, indole is toxic to *Blastocystis*. This indicates that high indole concentrations are toxic to *Blastocystis*, which is unsurprising, as indole is known to have an antimicrobial effect on other microorganisms [37,38]. Tryptophan was shown to increase both *Blastocystis* proliferation from a concentration of 2.5mM and viability from a concentration of 1mM. Tryptophan may also have a protective effect on *Blastocystis*, as it negated the ~50% population die-off caused by incubation in PBS (Fig 6A). *Bh*TnaA may be employed by the parasite to lower the level of indole in its local environment and increase the level of tryptophan, enabling greater survivability. This process may be especially relevant in the colon, where prokaryotes such as *E*. *coli* may generate localized areas of high indole concentration. Indole has also been associated with enteric pathogen virulence—enterohemorrhagic *E*. *coli* and *C*. *rodentium* downregulate virulence genes in response to indole within the intestinal lumen [39]. As *Blastocystis* behaves as a consumer of indole rather than a producer, it may be able to create localised ‘indole-free’ areas, increasing the virulence of some nearby bacteria species.

Figs 7 and 8 directly assessed the interaction between *Blastocystis* and *E*. *coli* in the context of tryptophan metabolism, when cultured in minimal media. *Blastocystis* ST7-B proved seemingly incapable of digesting tryptophan or synthesising indole in media supplemented with tryptophan (Fig 7A and 7B), while it was capable of producing a statistically significant increase in tryptophan concentration when cultured in media supplemented with indole (Fig 8A). This demonstrates the preference of *Bh*TnaA for indole-to-tryptophan metabolism. It was unexpected that *Blastocystis* did not synthesise any indole when it was supplemented with tryptophan–our indole tests show that *Blastocystis* has the ability to produce indole from tryptophan (Fig 3). This result may be due to other factors, such as the amount of indole produced being below the lower sensitivity bound of the test (3μM).

## Conclusions

Our results demonstrate that *Blastocystis* possesses a mutant form of the prokaryotic tryptophanase gene, *Bh*TnaA—a bacterial genetic homologue with divergent functionality from its canonically-understood form. This has implications for future *Blastocystis* research, as the possibility now exists for other genes in the organism to behave unexpectedly. This concept may have implications for model organism research as a whole–genes with known functions are generally assumed to have similar or identical roles when identified in similar organisms. We demonstrated that this may not be the case. There may be numerous examples of proteins with ‘known’ functions that operate in an opposing or entirely different fashion *in vivo*, especially in circumstances where HGT is known to have occurred. The inversion of the function of one of the initial enzymes of the *Blastocystis* tryptophan pathway is likely to influence elements of the entire pathway, and further work should be done to identify the extent of these changes, as well as their implications in the context of other microbiome species. It is also important to assess whether similar behaviour is observed in other subtypes of *Blastocystis*, and other isolates beyond ST7-B. It is also important to understand how *Blastocystis* interacts with other prokaryotes in its environment with regards to indole and tryptophan, for example whether hypothesised tryptophan cycle (Fig 9).

This study shows that *Bh*TnaA preferentially performs the reverse reaction of canonical tryptophanase genes, metabolising indole into tryptophan. This establishes *Blastocystis* as a potential producer of tryptophan within the gut. The majority of the body’s serotonin is produced from gastrointestinal tryptophan by enterochromaffin cells situated in the intestinal lumen [40]. One notable aspect of Irritable Bowel Syndrome is an association with serotonin [30,41,42]—specifically, elevated levels of serotonin have been found in the gut of individuals with IBS-like symptoms [31]. We hypothesise that an excessive infection of *Blastocystis* within a patient may lead to perturbed levels of tryptophan, which would be translated into perturbations in the levels of serotonin, kynurenic acid, and other downstream tryptophan byproducts, such as quinolinic acid. This may then cause symptoms analogous to those displayed by IBS patients.

## Supporting information

S1 Fig Flow cytometry gating strategy. R0 represents live cells. R1 represents dead cells. % Live cells calculated by R0/(R0+R1).
(TIF)

S2 Fig Effect of indole concentration on ST7B in IMDM-HS. Method was identical to that described in the Materials and Methods section, however initial seed density was 10^7^ cells, and media was IMDM-HS instead of PBS.
(TIF)
