# Supplementary figures and images for: Characterisation of novel functionality within the Blastocystis tryptophanase gene
Source: PLoS Negl Trop Dis. 2021 Sep 7;15(9):e0009730. doi: 10.1371/journal.pntd.0009730 (PMC8448343; doi:10.1371/journal.pntd.0009730)

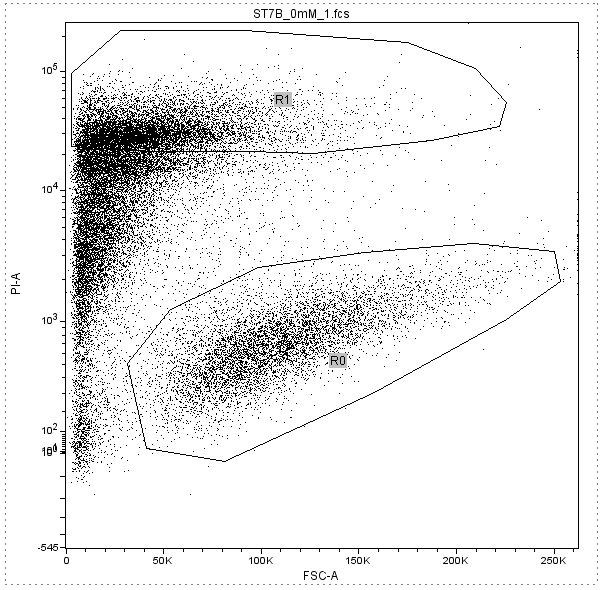

Supplement: S1 Fig — R0 represents live cells. R1 represents dead cells. % Live cells calculated by R0/(R0+R1). (TIF) [file pntd.0009730.s001.tif]

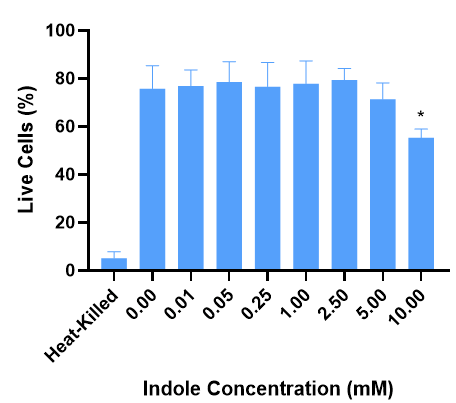

Supplement: S2 Fig — Method was identical to that described in the Materials and Methods section, however initial seed density was 107 cells, and media was IMDM-HS instead of PBS. (TIF) [file pntd.0009730.s002.tif]
